# Whistle variation in Mediterranean common bottlenose dolphin: The role of geographical, anthropogenic, social, and behavioral factors

**DOI:** 10.1002/ece3.6029

**Published:** 2020-02-05

**Authors:** Gabriella La Manna, Nikolina Rako‐Gospić, Gianluca Sarà, Federica Gatti, Silvia Bonizzoni, Giulia Ceccherelli

**Affiliations:** ^1^ MareTerra Onlus ‐ Environmental Research and Conservation Alghero Italy; ^2^ Blue World Institute of Marine Research and Conservation Veli Lošinj Croatia; ^3^ Dipartimento di Scienze della Terra e del Mare Università di Palermo Palermo Italy; ^4^ Università di Roma La Sapienza Roma Italy; ^5^ Dolphin Biology and Conservation Cordenons Italy; ^6^ OceanCare Wädenswil Switzerland; ^7^ Dipartimento di Chimica e Farmacia Università degli Studi di Sassari Sassari Italy

**Keywords:** acoustic behavior, geographic variation, Mediterranean Sea, *Tursiops truncatus*

## Abstract

The studies on the variation of acoustic communication in different species have provided insight that genetics, geographic isolation, and adaptation to ecological and social conditions play important roles in the variability of acoustic signals. The dolphin whistles are communication signals that can vary significantly among and within populations. Although it is known that they are influenced by different environmental and social variables, the factors influencing the variation between populations have received scant attention. In the present study, we investigated the factors associated with the acoustic variability in the whistles of common bottlenose dolphin (*Tursiops truncatus*), inhabiting two Mediterranean areas (Sardinia and Croatia). We explored which factors, among (a) geographical isolation of populations, (b) different environments in terms of noise and boat presence, and (c) social factors (including group size, behavior, and presence of calves), were associated with whistle characteristics. We first applied a principal component analysis to reduce the number of collinear whistle frequency and temporal characteristics and then generalized linear mixed models on the first two principal components. The study revealed that both geographic distance/isolation and local environment are associated with whistle variations between localities. The prominent differences in the acoustic environments between the two areas, which contributed to the acoustic variability in the first principal component (PC1), were found. The calf's presence and foraging and social behavior were also found to be associated with dolphin whistle variation. The second principal component (PC2) was associated only with locality and group size, showing that longer and more complex tonal sound may facilitate individual recognition and cohesion in social groups. Thus, both social and behavioral context influenced significantly the structure of whistles, and they should be considered when investigating acoustic variability among distant dolphin populations to avoid confounding factors.

## Graphical abstract

We investigated the factors influencing acoustic variability in the whistles of common bottlenose dolphin inhabiting two Mediterranean areas. The study revealed that both geographic distance/isolation and local environment are associated with whistle variations between localities. Both social and behavioural context influenced significantly the structure of whistles, and they should be considered when investigating acoustic variability among distant dolphin populations to avoid confounding factors.

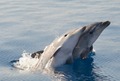

## 1 INTRODUCTION

The acoustic signals serve to mediate species and individual recognition, reproduction, resource, and territory defense in many diverse taxa, including insects, frogs, fish, birds, and mammals (Wilkins, Seddon, & Safran, 2012). The intraspecies geographic variation in acoustic communication is widespread in different taxa, and various conditions have been investigated to understand potential drivers of acoustic variability (Wilkins et al., 2012). For example, the acoustic signals in some species of birds, anurans, and mammals are dependent on the environment where the species live so that signal transmission is improved (the “acoustic adaptation hypothesis”, Morton, 1975; Wiley & Richards, 1982). Thus, the acoustic communication is correlated with environmental features, such as climate, habitat type, and soundscape (see Ey & Fischer, 2009 for a review). However, acoustic variability can also be caused by genetic differences, which in turn may be related to the geographical distance or isolation between populations (Amezquita et al., 2009; Campbell et al., 2010; Irwin, Thimgan, & Irwin, 2008). In addition, when acoustic behavior is mediated by vocal learning, cultural drift may be responsible for acoustic variability (Wilkins et al., 2012).

The variations in cetacean vocalization have been observed in many species, both at interspecific and intraspecific level (Azevedo, Oliveira, Dalla Rosa, & Lailson‐Brito, 2007; Hawkins, 2010; May‐Collado & Wartzok, 2008; Morisaka, Shinohara, Nakahara, & Akamatsu, 2005; Oswald, Barlow, & Norris, 2003; Papale et al., 2013; Rendell, Matthews, Gill, Gordon, & MacDonald, 1999; Wang, Würsig, & Evans, 1995). Nevertheless, only few attempts have been made to identify the different factors responsible for acoustic variation (Deecke, Ford, & Spong, 2000; Hatch & Clark, 2004; Hoffmann et al., 2012; Leao, Monteir‐Filho, & Silva, 2016; McDonald, Mesnick, & Hildebrand, 2006; Morisaka et al., 2005; Parks, Urazghildiiev, & Clark, 2009; Yurk, Barrett‐Lennard, Ford, & Matkin, 2002), apart from the investigation of phylogenetic distance between species (Ding, Würsig, & Evans, 1995; May‐Collado, Agnarsson, & Wartzok, 2007; Rendell et al., 1999) and the geographic distance between populations of the same species (Amano, Kourogu, Aoki, Yoshioka, & Mori, 2014; Ansmann, Goold, Evans, Simmonds, & Simon, 2007; Azevedo & Van Sluys, 2005; Azzolin, Papale, Lammers, Gannier, & Giacoma, 2013; Baron, Martinez, Garrison, & Keith, 2008; Bazua‐Duran & Au, 2004; Delarue, Todd, VanParijs, & Di Iorio, 2009; Rendell & Whitehead, 2005; Rossi‐Santos & Podos, 2006; Wang et al., 1995).

The bottlenose dolphin, *Tursiops* spp., live in complex fission–fusion societies (Connor, Wells, Mann, & Read, 2000; Mann, Connor, Barre, & Heithaus, 2000) and have developed frequency‐modulated, narrow‐band signals, called whistles, used for individual recognition, contact maintenance, and group coordination (Janik & Sayigh, 2013; MacFarlane et al., 2017). Many studies have found acoustic variation between different populations of bottlenose dolphin (Azevedo et al., 2007; Hawkins, 2010; Jones & Sayigh, 2002; La Manna, Rako‐Gospić, Manghi, Picciulin, & Sarà, 2017; May‐Collado & Wartzok, 2008; Morisaka et al., 2005; Papale et al., 2013; Wang et al., 1995), but understanding such variations without disentangling the concurrent effects of different factors shaping dolphin acoustic behavior may be limiting and frustrating (Gridley, Elwen, Rashley, Badenas Krakauer, & Heiler, 2017; Heiler, Elwen, Kriesell, & Gridley, 2016; May‐Collado & Quiñones‐Lebrón, 2014; Sayingh, 2014). Because the soundscape of a given environment may change over time, variations in dolphin whistles may be the response to varying background noise levels, for example, to facilitate signal transmission and have an effective communication (Ansmann et al., 2007; May‐Collado & Wartzok, 2008; Papale, Gamba, Perez‐Gil, Martin, & Giacoma, 2015). The changes in vocal behavior may also depend on factors, including group behavior and the occurrence of anthropogenic stressors (Gridley et al., 2017; Hawkins & Gartside, 2009; La Manna, Manghi, Pavan, Lo Mascolo, & Sarà, 2013; Marley, Salgado, & C.P., Erbe, C. and Parnum, I.M., 2017; Rako‐Gospić & Picciulin, 2016; Romeu, Cantor, Bezamat, Simões‐Lopes, & Daura‐Jorge, 2017). The features, such as group size and composition, are also known to influence the acoustic properties of whistles and contribute to their variation (Heiler et al., 2016; Quick & Janik, 2008). Overall, because of the ability for vocal learning and the vocal plasticity of bottlenose dolphin, the structure and characteristics of whistles are influenced and shaped by social and environmental factors. For example, variations in individual‐specific signature whistles—the most commonly used whistles (Cook, Sayigh, Blum, & Wells, 2004; Janik & Sayigh, 2013)—may occur when individual bottlenose dolphins imitate the signature whistle of a conspecific (King, Sayigh, Wells, Fellner, & Janik, 2013; Sayigh & Janik, 2010). Thus, dolphins in nearby areas may influence each other's whistles through mimicry (Janik & Slater, 2000; Wang et al., 1995). Furthermore, whistle similarity has been related to the strength of individual social relationships. For example, closely associated males share more whistle types than nonassociated animals (Smolker & Pepper, 1999; Watwood, Tyack, & Wells, 2004).

The acoustic variability (measured through changes in whistle frequencies and time characteristics) has been already described among *Tursiops truncatus* (hereafter referred to as bottlenose dolphin) populations of the western (Sicily and Sardinia, Italy) and eastern (Croatia) Mediterranean (La Manna et al., 2017). In the present work, the factors associated with bottlenose dolphin acoustic variability between two populations, located (2000 km apart) in Sardinia Island (western Mediterranean Sea) and Croatia (northern Adriatic Sea), were identified. To achieve this aim, the influence of anthropogenic conditions (noise levels and boat presence) and dolphin socio‐behavioral context (dolphin behavior, group size, and the occurrence of calves within the group) on the whistle structure was considered separately. The identification of the factors associated with the acoustic variability between the two populations will draw the attention of researchers on what kind of features dominate the whistle structure of bottlenose dolphin—whether noise and boat presence can affect their communication more than the natural conditions. The results may contribute to providing relevant evidence for managing human activities in bottlenose dolphin areas.

## 2 METHODS

### 2.1 Study areas

**Figure 1:**
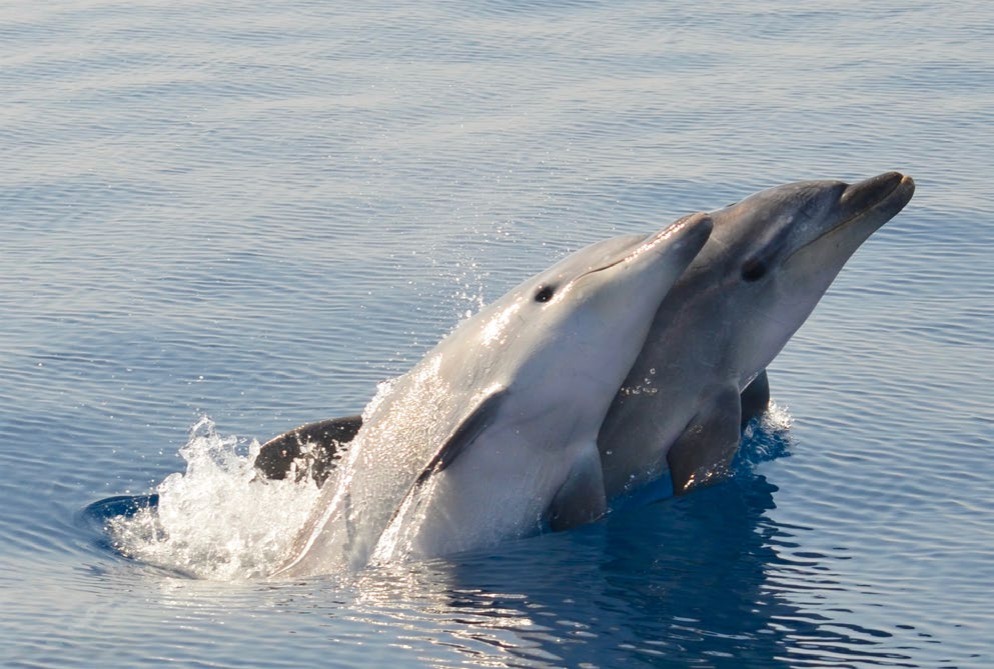
The bottlenose dolphin (*Tursiops truncatus*)

**Figure 2:**
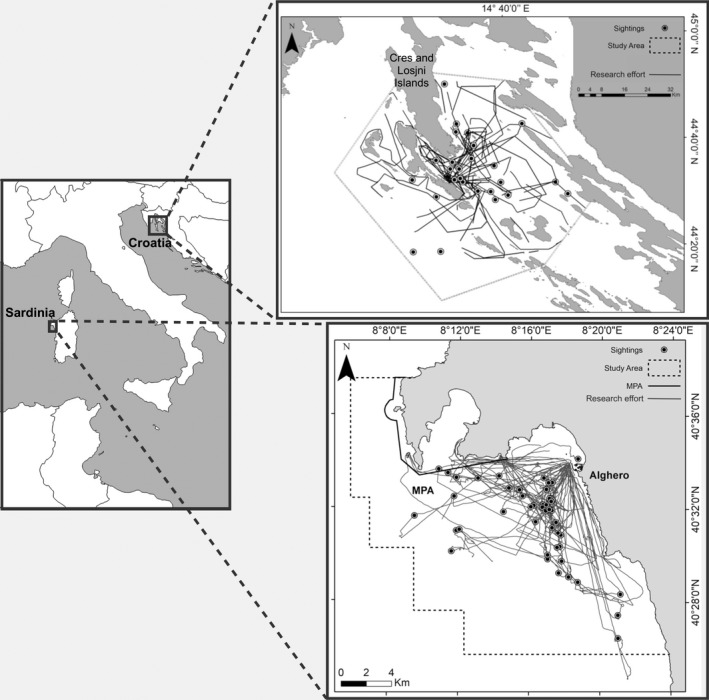
Map of the two study areas in the Mediterranean Sea: Sardinia and Croatia. Dotted lines indicate the size of the study areas. Gray lines and black points indicate the tracks and dolphin sightings (only those included in the analysis are showed)

The Sardinia and Croatia bottlenose dolphin (Figure 1) populations are separated geographically by the Italian peninsula (Figure 2), and based on their home range estimates (that do not overlap), they can be considered two distinct populations. In fact, the home ranges for the resident individuals of the two populations were estimated to be 1,294.3 km^2^ (mean 95% fixed‐kernel density estimator on 44 individuals—Rako‐Gospić et al., 2017) and 330 km^2^ (mean 95% fixed‐kernel density estimator on 17 individuals—La Manna & Ronchetti, 2018) in Croatia and Sardinia, respectively.

The acoustic recordings in Sardinia were made off the main harbor of Alghero (40.5580°N, 8.3193°E) in an area of about 450 km^2^ (Figure 2), that extends beyond the currently known home range of the population. The area includes a gradually graded rocky bottom, *Posidonia oceanica* meadows, and a detrital bottom with a depth not exceeding 115 m. The boat traffic during summer increases considerably because sea‐related tourism is one of the main economic activities of the surrounding communities (La Manna, Manghi, Perretti, & Sarà, 2016). A bottlenose dolphin population (121 photo‐identified individuals) inhabits these waters, where at least 50% of them show a high level of site fidelity (La Manna & Ronchetti, 2018). Although the majority of these animals were sighted repeatedly every year and in different seasons, the population seems neither closed nor isolated. In fact, between 2012 and 2018, the discovery curve of the photo‐identified dolphins never reached a plateau because of the regular entrance of new individuals (La Manna & Ronchetti, 2018).

The acoustic recordings in Croatia were undertaken in an area of about 2000 km^2^, that extends beyond the currently known home range of the population, around the islands of Cres and Lošinj (Figure 2). These waters are characterized by numerous uninhabited small islands and islets, steep rocky shores, muddy sea bottoms, limestone reefs, and sea depths that do not exceed 90 m of depth. The islands attract a large number of tourists each year, particularly during summer when many of them reach these sites by leisure boats (Rako‐Gospić et al., 2013; Rako‐Gospić & Picciulin, 2016). This region is part of the home range of about 200 common bottlenose dolphins. Based on a photo‐identification study, 184 individuals (95% CI = 152–250; CV = 0.17) were estimated by means of mark–recapture methods and the Mth estimator of Chao for closed populations (Pleslić et al., 2015; Rako‐Gospić et al., 2017). The high sighting frequency and regular resightings of known individuals from year to year indicate their long‐term fidelity to this specific region. Because of its importance as a habitat for this resident bottlenose dolphin population, the Cres‐Lošinj area was designated as a site of community importance (SCI), part of the European Union NATURA 2000 ecological network (Cres and Lošinj SCI, HR3000161), in December 2014.

### 2.2 Field methodology

The data were collected during dedicated boat surveys in spring and summer between 2015 and 2018; overall, 36 and 52 surveys were done in Croatia and Sardinia, respectively. The daily surveys were conducted using a 5.8 m RIB powered by a four‐stroke 90 HP outboard engine in Croatia and a 9.7 m motor boat powered by a 270 HP inboard engine in Sardinia. With the aim to homogeneously cover the study area, routes were designed with a generally perpendicular direction with respect to the coast and depth contours. The experienced observers scanned the sea surface during daylight, in good sea conditions (Douglas sea state < 2 and Beaufort wind force < 2), with a visibility of over 3 nautical miles, and a boat speed between 10 and 30 km/h. The navigation routes were interrupted in case of sighting or when sea conditions deteriorated. A dolphin sighting was defined as an observation of one dolphin or a group of dolphins (defined as all individuals within visual range that were in apparent association, engaged in the same activity or moving in the same direction; Shane, 1990). During each sighting, data on dolphin group size and age class were recorded by two independent observers. The calves were defined as dolphins of no more than two‐thirds the length of an adult (Shane, 1990). The individuals not belonging to the calf class were defined as adults/subadults. The adults were fully grown individuals, generally 2.5–3 m long, while subadults were not yet fully grown but were larger than the calves and did not travel in the typical calf position alongside an adult individual. The identity of individuals in the group was determined using standard photo‐identification techniques (Würsig & Jefferson, 1990), using a Nikon D7000 and a Sony alpha 65 cameras equipped with 70–300 and 18–250 mm lenses in Sardinia and a Canon EOS 6D equipped with a 70–200 mm lens in Croatia. Each image was evaluated for photographic quality and grade of distinctiveness of the fin following Ingram (2000). Then, two researchers independently matched the photographic datasets to avoid misidentification (Pleslić et al., 2015; Pulcini, Pace, Manna, Triossi, & Fortuna, 2013).

The behavior and acoustic data were collected 20 min following the first approach, allowing the animals to habituate to the research boat presence. The surface behavioral state was recorded by following continuous focal group sampling (Altmann, 1974; Mann, 2000). The observations lasted between 20 and 60 min. The behavioral states assigned included one of the following mutually exclusive categories (Lusseau, 2003; Shane, 1990): (a) foraging (animals usually dispersed, frequent direction changes, dive intervals longer than 3 min, fish chases at the surface, birds often in attendance); (b) traveling (consistent directional movement of dolphins, with regular surfacing, often much splashing); (c) socializing (interactive events observed, such as body contacts, pouncing and hitting with tail, chases, aerial events, no directed movements, and variable dive intervals); (d) milling (no net movement, individuals surface facing different directions, dive intervals variable but short); and (e) resting (slow movement of dolphins, no splashing, closely associated, short and synchronous dive intervals). If members of the group displayed more than one category, the predominant one (performed by more than 50% of the group members) was recorded (Mann, 1999). To avoid potential bias related to group composition and assigning an incorrect behavioral state to the recorded acoustic behaviors, the data collected when animals in the focal groups performed different behavior were not considered in the analysis.

Concurrently to the collection of surface behavior data, the acoustic recordings were gained by means of a hydrophone. In Sardinia, a Sensor Technology SQ26‐08 omnidirectional hydrophone (sensitivity −168.8 dB re 1 V/µPa; flat frequency response from 100 Hz to 30 kHz, ±3 dB), with a bandwidth between 20 Hz and 50 kHz, was lowered to a 5–10m depth and connected to an M‐Audio MicroTrack II recorder or a ZOOM recorder (data format 24 bit WAV, sampling rate 96 kHz). Before each recording, the recording system was calibrated by applying a signal of 100 mV RMS at 2 kHz to the transducer input of the system by means of a signal generator. In Croatia, a RESON TC 4,032 omnidirectional hydrophone (sensitivity − 170 dB re 1 V/µPa; flat frequency response from 10 Hz to 80 kHz, ± 2.5 dB), with a bandwidth between 5 Hz and 120 kHz, was lowered at approximately 5 m of depth and connected to a SOUNDDEVICES 702 high‐resolution digital audio recorder (data format 24‐bit WAV, sampling rate 192kHz), calibrated with a signal of 100 mV RMS at 2 kHz. In order to minimize mechanical noise, such as flow and cable strumming noise, which may be induced when low‐frequency ambient noise is measured by cabled hydrophone, we recorded only in good sea and wind condition (Douglas sea state < 2 and Beaufort wind force < 2), in absence of wave motion. Furthermore, the engine and the instruments on board were switched off and the boat was still during all the recordings.

The behavioral categories have been assigned to each whistle according to surface behavior data collected as described above. To ensure the correct behavioral category assignment to each whistle, data collected in the presence of more than one group of dolphins within the visual range of the observers were discarded. Only whistles with the highest signal to noise ratio (strongly suggesting a close proximity of the recorded individuals) were analyzed (Heiler et al., 2016; Marley et al., 2017). During the behavioral and acoustic data collection, the research boat remained between 20 and 100 m away from the dolphins with the engine off. The number and type of boats present (except for those stationary with the engine off) within 500 m of the focal group were also recorded. The maximum distance of 500 m was chosen because it should be the approximate distance at which a boat noise is considerably higher than the background noise (following Sara' et al., 2007), although this noise may change relevantly depending on the type and speed of the boat and the environmental conditions.

### 2.3 Whistle and sea ambient noise analysis

A whistle was defined as a narrow‐band tonal signal lasting 0.1 s or more, with at least part of the fundamental frequency above 3 kHz (Gridley, Berggren, Cockcroft, & Janik, 2012; Heiler et al., 2016; Kriesell, Elwen, Nastasi, & Gridley, 2014).

This strict definition is useful to distinguishe whistles from other narrow‐band sounds produced by bottlenose dolphins (Simard et al., 2011; van der Woude, 2009). The whistles are distinguished as signature and nonsignature whistles (Caldwell, Caldwell, & Tyack, 1990; Janik, Dehnhardt, Todt, 1994; Cook et al., 2004; Janik & Sayigh, 2013). The nonsignature whistles, also known as variant whistles, are not characterized by individually distinctive frequency‐modulated patterns (Caldwell & Caldwell, 1979; Watwood et al., 2004; King et al., 2013), and their function remained poorly understood (Janik & Slater, 2000; Tyack, 1986). A signature whistle is defined as “a learned, individually distinctive whistle type in a dolphin's repertoire that broadcasts the identity of the whistle owner” (Janik & Sayigh, 2013). Thus, signature whistles are characterized by the same frequency modulation pattern (called contour), at frequencies ranging between 1 and 38 kHz (Boisseau, 2005; Hiley, Perry, Hartley, & King, 2016; May‐Collado & Wartzok, 2008), with a duration between 0.1 and 4.0 s (Buckstaff, 2004). The signature whistles can be produced in loops (repetitions of the same elements), usually separated by intervals less than 250 ms (Esch, Sayigh, Blum, & Wells, 2009), and can also have an introductory and/or final loop (Janik & Sayigh, 2013) distinct from the central pattern. We considered any single‐ or multiple‐loop whistle, connected or disconnected, as a unit of analysis (Esch et al., 2009).

Each whistle, recognized as a signature whistle by the observer following the SIGnature IDentification method (SIGID; see Janik & Sayigh, 2013), was considered just once in the analysis. This rule was applied to reduce the risk of collecting whistles from the same individual (pseudoreplication), thus introducing a bias in the sample because of the repetition of whistles characterized by the same contour (La Manna, Rako‐Gospić, Manghi, & Ceccherelli, 2019; La Manna et al., 2017). Following a method already applied in other studies (Heiler et al., 2016; La Manna et al., 2013, 2019; Marley et al., 2017), all whistles were graded on the basis of their signal to noise ratio (SNR) as (a) score 1 (faint whistle with the entire contour not clearly visible on the spectrogram or overlapping with other sounds); (b) score 2 (whistle clearly visible from its start to its end); and (c) score 3 (prominent and dominant whistle). Only whistles scoring 2 or 3 were analyzed further. The duration; minimum, maximum, start, and end frequencies; frequency range; and the number of inflection points (Table 1; La Manna et al., 2013, 2017; Papale et al., 2013) were measured by visual inspection of the spectrogram under Raven Pro 1.4 [Bioacoustics Research Program, 2014—512/1024‐point fast Fourier transform (FFT) and frame length, Hamming window, 50% overlap, Fs. = 48 kHz; licensed to Gabriella La Manna].

**Table 1:** Whistle parameters measured on the spectrogram (manually or automatically by Raven Pro 1.4 software)

Parameter	Unit	Description
Start frequency	Hz	The frequency measurement at the start of the whistle.
End frequency	Hz	The frequency measurement at the end of the whistle.
Min frequency	Hz	The lower frequency limit of the selection box.
Max frequency	Hz	The upper frequency limit of the selection box.
Frequency range	Hz	Total bandwidth, calculated by max frequency minus min frequency.
Duration	Sec	Total duration, calculated by end time minus start time.
Number of inflection points	–	The number of inflection points defined as the change from positive to negative or negative to positive slope in the contour.

A selection of 5 s, immediately before each whistle of good quality (score 2 or 3), was analyzed by means of PAMGuide. The PAMGuide is a template code provided in R (Merchant et al., 2015), able to perform the signal processing steps required for the calibration procedure to obtain absolute sea ambient noise (SAN) levels. For the purposes of this study, SAN is defined as “all sound (both natural and anthropogenic) except that resulting from the deployment or recovery of the recording equipment” (Robinson, Lepper, & Hazelwood, 2014). The choice of 5 s was a compromise between the supposed minimum time required to adjust a vocal emission to background noise level in mammals (2 s; Gillam, Ulanovsky, & McCracken, 2007; Hase, Miyamoto, Kobayasi, & Hiryu, 2016) and the maximum duration of whistle recorded in the present study (4.3 s). The SAN was measured as sound pressure level (SPL), and each selection was described in terms of the broadband between 2 and 20 kHz, corresponding to the frequency range of the majority of bottlenose dolphin whistles (van Ginkel, Becker, Gowans, & Simard, 2017; Lammers & Oswald, 2015; Rako‐Gospić et al., 2013) and 1/3 octave bands between 125 Hz and 20 kHz. The 125 Hz band is one of the indicators of the Marine Strategy Framework Directive (MSFD) for “continuous low‐frequency sound” and is a good predictor of boating noise (Picciulin et al., 2015).

### 2.4 Statistical analyses

The descriptive statistics (mean, standard deviation, CV = coefficient of variation and range) about the acoustic characteristics of all whistles in the dataset were used to describe the intra‐ and interpopulation variability. Before any analysis, data exploration was carried out following Zuur, Ieno, Walker, Saveliev, and Smith (2009).

We were interested in disentangling which variables influenced the geographic variation of whistles between Sardinia and Croatia. Thus, we preliminarily tested if the sea ambient noise levels and group size were associated with the factor locality, to explore ecological and acoustical environment differences. We applied three generalized linear models (GLM) using a negative binomial distribution (to account for overdispersion), where SAN levels (in the three octave bands, 125 Hz, 2000 Hz, and 20 khz) were the dependent variables and locality was the predictive variable. The model diagnostics in R was used to verify the appropriateness and assumptions for each model, and the analysis of deviance (MASS package in R) was used to assess the significance of the predictors (Venables & Ripley, 2002).

Then, a principal component analysis (PCA) was used to reduce the seven acoustic whistle characteristics into two independent variables, after the assumptions (linear relation between variables, sampling adequacy, and presence of outliers) had been verified. Only the first two components (that together explained 71% of the total variance, see the Results Section) were retained because eigenvalues for the remaining five components were all < 1 (Kaiser's criterion). To perform PCA, the function prcomp of the R package Rstats (R Core Team, 2015) was used. The association between the two PCs and the geographical (locality), anthropogenic (noise levels in the three bands and boat presence), social (group size and calf presence), and behavioral variables was tested using a generalized linear mixed model (GLMM—following Jansen, Plath, Brusquetti, & Ryan, 2016; Lee, Shaner, Lin, & Lin, 2016) with a gaussian distribution. The GLMMs are an extension of generalized linear models that allow for the inclusion of random effects, by modeling the covariance structure that is generated by the grouping of data (Zuur et al., 2009). They are very useful when the data are not independent, for example, when a variable is measured more than once from the same individuals. Because the whistles recorded within the same group are likely to be more related to each other than the whistles recorded from different groups, we considered the group as a random factor in the models. We first explored covariates to check for multicollinearity with pair plots, and variance inflation factors (VIFs) were calculated. All octave band noise levels were highly correlated with the exception of those centered at 125 Hz, 2000 Hz, and 20,000 Hz (VIFs < 2); thus, only these bands were included in the models. The lowest bands (125 Hz) were used because of its relevance as indicator of the Marine Strategy Framework Directive (MSFD). Besides, 125 Hz band was correlated to the 500 Hz and 1,000 Hz bands, which represents the lowest limits of the whistles recorded in the two areas. With the aim to investigate the effect of the acoustic environments and boat presence separately from that of the social and behavioral context of the two areas, two kinds of models were built: One was performed to test the association between PCs and noise levels and boat presence as fixed terms (noise levels in the three octave bands as covariates and boat presence as a factor) and the other to the test the association between PCs and social and behavioral variables as fixed terms (group size as a covariate, and calf presence and behavioral states as factors). Since resting and milling had a negligible sample size, the only behavioral states included in the models were feeding, traveling, and socializing. Because one of the main aims of the analysis was to assess the effect of locality, in both models we included the interaction between locality and the fixed terms. We followed a forward selection procedure to select the best models, based on the Akaike's information criterion (AIC) and likelihood ratio tests (Zuur et al., 2009). Afterward, the best model was validated by means of graphical inspection of residuals (i.e., residuals vs. fitted values plots to verify homogeneity; Q–Q plots of the residuals for normality; and plots of residuals vs. each explanatory variable to check for independence). To perform the GLMMs, the function lme of the R package nlme (Pinheiro, Bates, DebRoy, & Sarkar, 2018) was used.

### 2.5 Ethical note

The study was entirely observational, and no special permit was necessary. Special care was taken when approaching the animals to reduce any disturbance and alteration of the natural behavior.

## 3 RESULTS

Based on the photo‐identification data, we identified 72 individuals in Sardinia and 256 individuals in Croatia, differently associated within the groups. Particularly, in Sardinia 30% of the individuals were sighted only once, 44% from 2 to 6 times, 15% from 7 to 11 times, and 11% from 12 to 17 times, while in Croatia 48% of the individuals were sighted only once, 49% from 2 to 6 times, and 3% from 7 to 8 times. The group size in Sardinia ranged between 2 and 16 animals, with a mean of 7.6 (*SD* ± 3.2) and a median of 8, while in Croatia it ranged between 2 and 46, with a mean of 22.1 (*SD* ± 12.3) and a median of 23. GLM associated group size to locality (Table 4). In Sardinia, 65% of whistles were recorded in absence of calves; 53% of them were recorded while dolphins were socializing, 30% while they were traveling, and 17% while they were foraging. In Croatia, 87% of whistles were recorded in presence of the calves; 65% of them were recorded while dolphins were traveling, 20% while they were foraging, and 15% while they were socializing. Among all the whistles, 56% and 66% were recorded in presence of boats, in Sardinia and Croatia, respectively.

### 3.1 Acoustic recordings

In Sardinia, a total of 27 hr 49 min of recordings were collected over 60 days, from which we extracted a total of 1980 whistles belonging to 60 different groups. In Croatia, a total of 28 hr 29 min of recordings were collected over 68 days, during which 89 dolphin groups were sighted, producing in total 927 whistles. From the whole dataset, stereotyped whistles were considered only once, and only whistles scored as 2 or 3 were analyzed further, reducing the initial database as follows: 456 whistles from 52 groups and 361 whistles from 36 groups in Sardinia and Croatia, respectively, with a general mean values of whistles higher in Croatia. Moreover, CVs were higher for duration and number of inflection points across all areas, and higher for all characteristics in Sardinia compared to Croatia, with the exception of number of inflection points and frequency range (Table 2).

**Table 2:** Descriptive statistics of whistle parameters from the two study areas (Sardinia and Croatia)

	Duration	*N* inflection points	Frequency range	Min frequency	Max frequency	Start frequency	End frequency
Sardinia (*n* = 456)							
Mean ± *SD*	0.81 ± 0.59	1.86 ± 2.14	6.56 ± 3.79	7.05 ± 3.34	13.61 ± 4.93	8.36 ± 4.08	11.12 ± 5.03
Range	0.03–3.93	0–13	0.17–30.86	0.41–22.19	1.34–35.82	0.60–25.21	0.35–27.99
CV	72.84	115.00	57.77	47.38	36.22	48.80	45.23
Croatia (*n* = 361)							
Mean ± *SD*	0.84 ± 0.53	1.38 ± 1.70	8.63 ± 6.70	6.89 ± 2.00	15.52 ± 3.92	9.10 ± 3.74	12.42 ± 5.00
Range	0.03–3.29	0–11	1.07–20.29	1.82–13.91	4.46–27.42	1.82–21.43	3.57–27.42
CV	63.09	123.18	77.63	29.02	25.26	41.10	40.26

The SAN levels were higher in Sardinia compared to Croatia, both in the 125 Hz and 20 kHz bands (Table 3). Accordingly, GLM results showed that SAN levels were associated with the factor locality, both in the 125 Hz and in the 20 kHz bands, while no influence was found on the SAN level in the 2 kHz band (Table 4).

**Table 3:** Descriptive statistics of the three‐band noise levels in Sardinia and Croatia

	SPL 125 Hz (dB re 1 µPa rms)	SPL 2 kHz (dB re 1 µPa rms)	SPL 20 kHz (dB re 1 µPa rms)
Sardinia (*n* = 456)			
Mean ± *SD*	95 ± 9	98 ± 9	103 ± 7
Range	84–135	84–141	92–127
Croatia (*n* = 361)			
Mean ± *SD*	89 ± 11	99 ± 12	87 ± 10
Range	61–126	68–134	61–123

**Table 4:** Results of the four GLM (negative binomial distribution) run on noise levels in the three bands (125 Hz, 2 kHz, and 20 kHz) and group size as a function of locality (Sardinia vs. Croatia)

	*df*	SPL 125 Hz				SPL 2 kHz			
		Deviance	Res. *df*	Dev. Res	Pr (>Chi)	Deviance	Res. *df*	Dev. Res	Pr(>Chi)
Null			816	857.54			816	808.02	
Locality	1	52.35	815	805.19	**<.0001**	1.1332	815	806.89	.2871

	*df*	SPL 20 kHz				Group size			
		Deviance	Res. *df*	Dev. Res	Pr (>Chi)	Deviance	Res. *df*	Dev. Res	Pr(>Chi)
Null			816	1,147.89			816	1654.87	
Locality	1	495.91	815	651.99	**<.0001**	870.69	815	784.18	**<.0001**

Note

Null model contain only the intercept as a parameter.

Abbreviations: *df*: degrees of freedom; Res. *df*: residual degrees of freedom; Dev. Res: residuals deviance.

Bold values are statistically significant.

**Figure 3:**
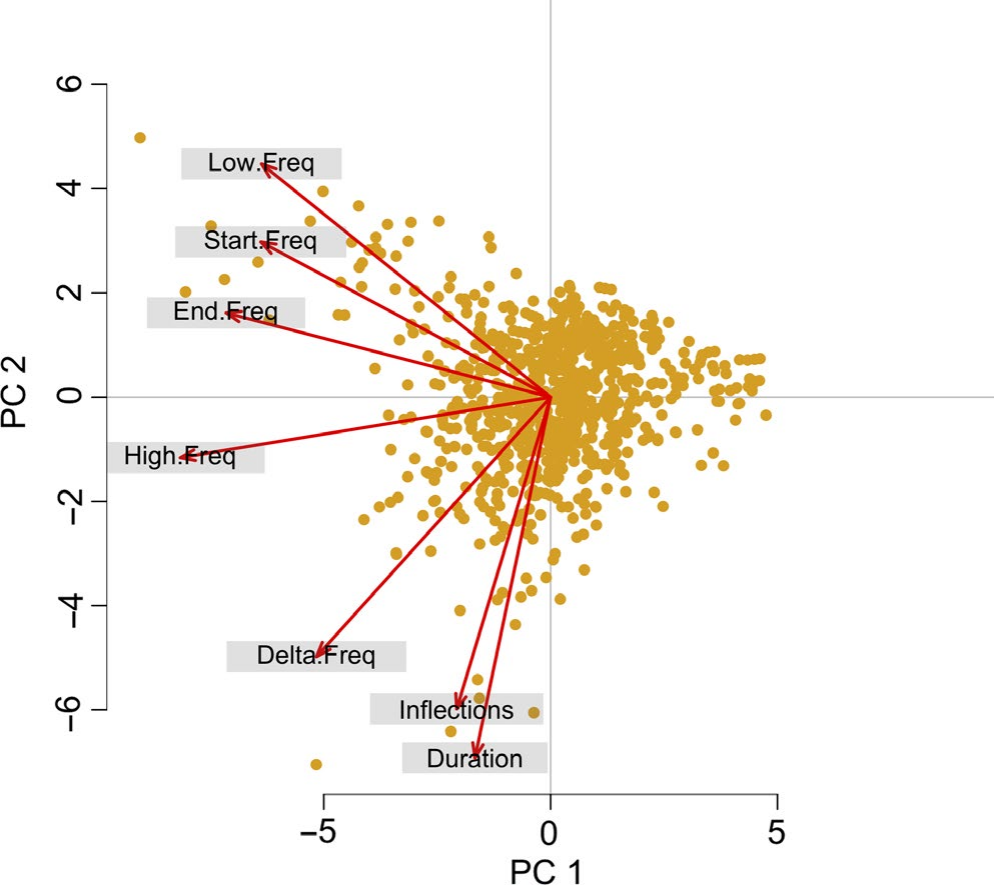
PCA biplot displays the information on correlation among variables. The directions of the arrows show the relative loadings of the parameters on PC1 and PC2

The first principal component (PC1) explained 45% of the variance and was negatively correlated with min, max, start, and end frequencies, characteristics that reflect the spectral properties of the whistles, while the second principal component (PC2) explained 26% of the variance, and it was negatively correlated with duration, number of inflection points, and frequency range, characteristics that reflect the temporal property and frequency modulation of the whistles (Figure 3, Table 5). We used GLMMs on PC1 and PC2 to investigate the effect of SAN and boat presence and socio‐behavioral variables with separated models. On the basis of the graphic validation of the models, no problems were found (Appendix S1: ES1, ES2, ES3, and ES4).

**Table 5:** Loadings of the first two principal components explained 71% of the total variance of the whistle acoustic parameters

Acoustic parameters	Principal component	
	PC1	PC2
Min frequency	**−0.41759**	0.37637
Max frequency	**−0.53485**	−0.09759
Start frequency	**−0.41858**	0.25043
End frequency	**−0.46871**	0.13587
Frequency range	−0.33811	**−0.41878**
Number of inflection points	−0.13565	**−0.58219**
Duration	−0.10926	**−0.50331**

Bold values are statistically significant.

### 3.2 Influence of noise and boat presence on whistle structure

**Figure 4:**
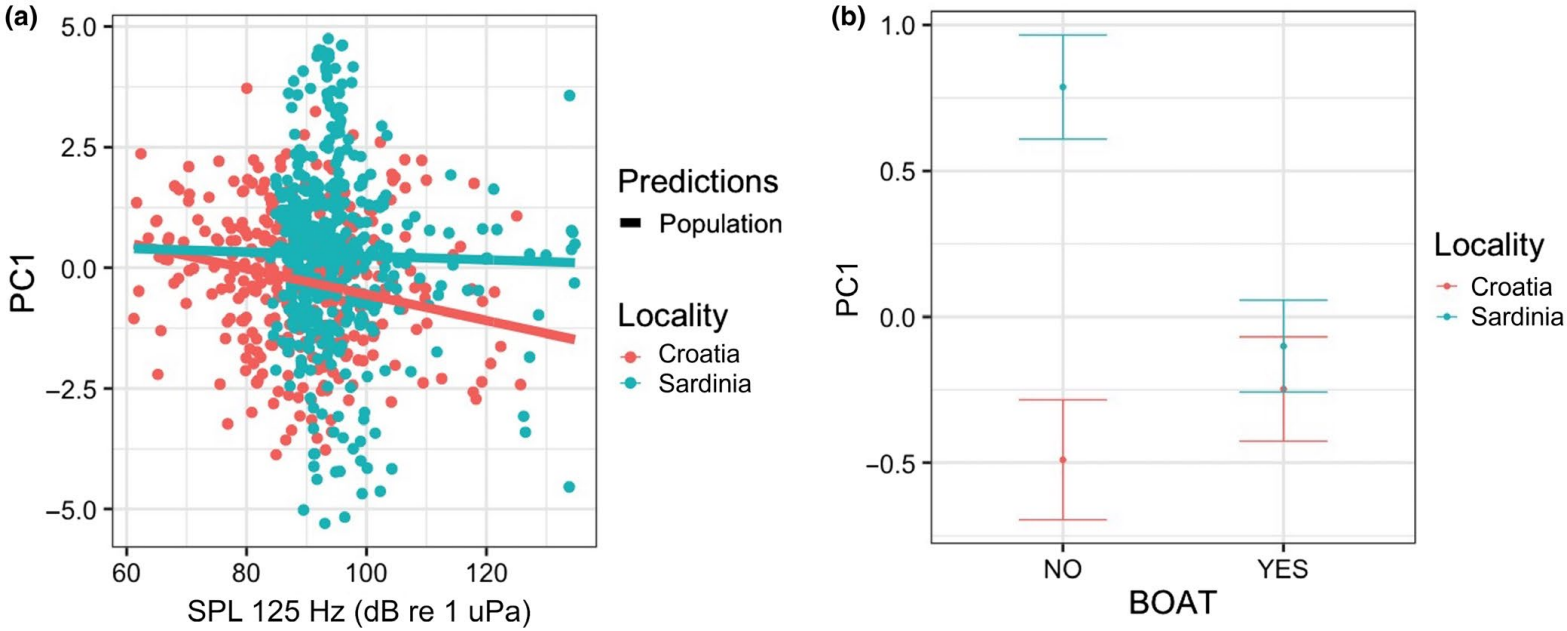
Interaction effect between (a) locality and SPL 125 Hz (dB re 1 µPa) and (b) locality and boat on the spectral property of the whistles (PC1) as predicted by the GLMM (elaborated with the package “nlme” in R)

**Figure 5:**
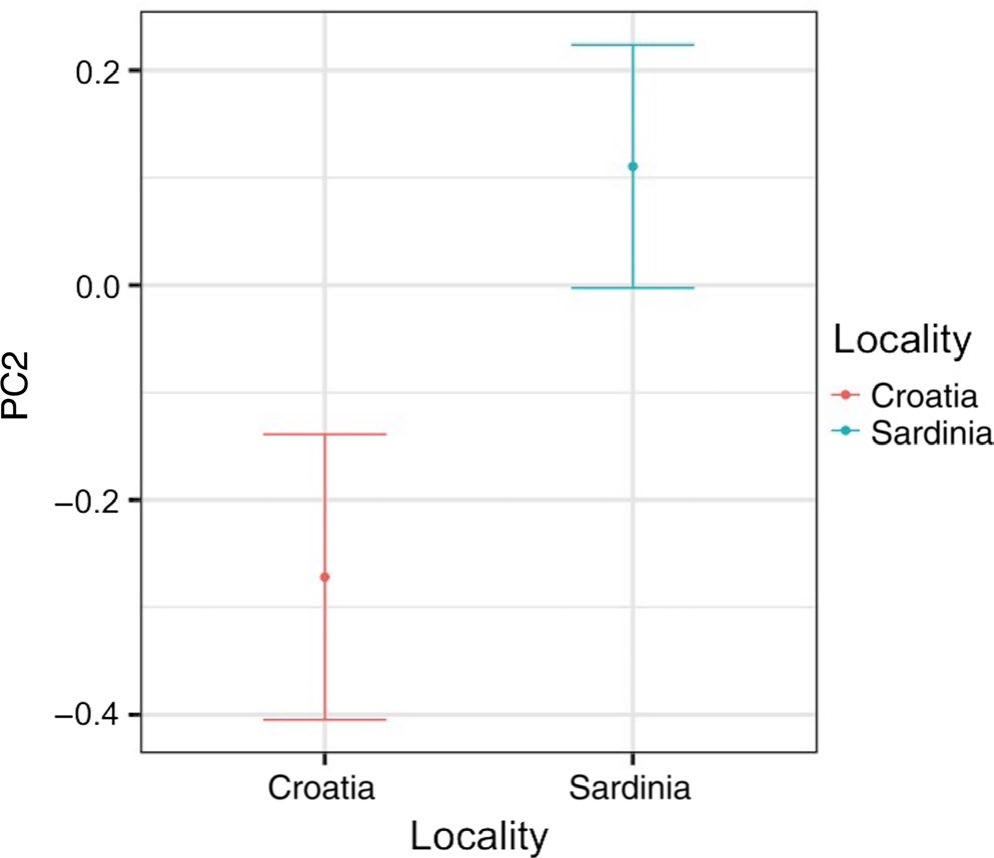
Effect of locality on the temporal property and modulation of the whistles (PC2) as predicted by the GLMM (elaborated with the package “nlme” in R)

The PC1 was significantly associated with the interaction between SPL in the 125 Hz band and locality and with the interaction between boat presence and locality (Table 6). In particular, PC1 decreased with the increasing noise in the 125 Hz band mainly in Croatia and decreased in the presence of boats in Sardinia (Figure 4). Thus, because the PC1 and min, max, start, and end frequencies were negatively correlated, the latter characteristics tend to increase with the increasing noise in Croatia and in presence of boats in Sardinia. The PC2 correlated only to locality (Table 7) and was higher in Sardinia compared to Croatia (Figure 5). In particular, duration and frequency range were lower in Sardinia.

**Table 6:** Generalized linear mixed‐effect model (GLMM) with anthropogenic explanatory variables

Effect				
Fixed effects	Value	*SE*	*t*‐value	*p*‐value
(Intercept)	2.01899	0.78980	2.55633	.0108
Locality	−1.41438	1.16559	−1.21345	.2253
SPL 125 Hz	−0.02706	0.00835	−3.23997	.0012
Boat	0.24263	0.22376	1.08435	.2786
SPL 125 Hz: Locality	0.02903	0.01223	2.37459	.0178
Boat: Locality	−1.13002	0.30166	−3.74594	.0002

*Random effects*	*SD* (Intercept)	Residual		
Group (Intercept)	0.7565	1.4583		

ANOVA between models	*df*	AIC	Likelihood ratio test	*p‐*value
Full model	12	3,037.322	−1506.661	
Best model	8	3,030.115	−1507.057	.9393

Note

The upper section shows the significant effects of the assessed explanatory variables on PC1. Value, standard errors (*SE*), *t*‐values, and significance level (*p*‐value) for variables retained in the best model are provided for fixed effects (explanatory variables), while estimates of the standard deviations (*SD*) are reported for random effects (group). The lower section presents the results of the model selection and significance of dropping the nonsignificant variables from the full model to obtain the best model. SPL = sound pressure level, “:” = interaction.

**Table 7:** Generalized linear mixed‐effect model (GLMM) with anthropogenic explanatory variables

Effect				
Fixed effects	Value	*SE*	*t*‐value	*p*‐value
(Intercept)	−0.27195	0.13287	−2.04678	.04100
Locality	0.38256	0.14343	2.66727	.00780

Random effects	*SD* (Intercept)	Residual		
Group	0.677	1.2118		

ANOVA between models	*df*	AIC	Likelihood ratio test	*p‐*value
Full model	12	2,741.467	−1358.734	
Best model	4	2,730.563	−1361.282	.7473

Note

The upper section shows the significant effects of the assessed explanatory variables on PC2. Value, standard errors (*SE*), *t*‐values, and significance level (*p*‐value) for variables retained in the best model are provided for fixed effects (explanatory variables), while estimates of the standard deviations (*SD*) are reported for random effects (group). The lower section presents the results of the model selection and significance of dropping the nonsignificant variables from the full model to obtain the best model.

### 3.3 Influence of socio‐behavioral variables on whistle structure

**Figure 6:**
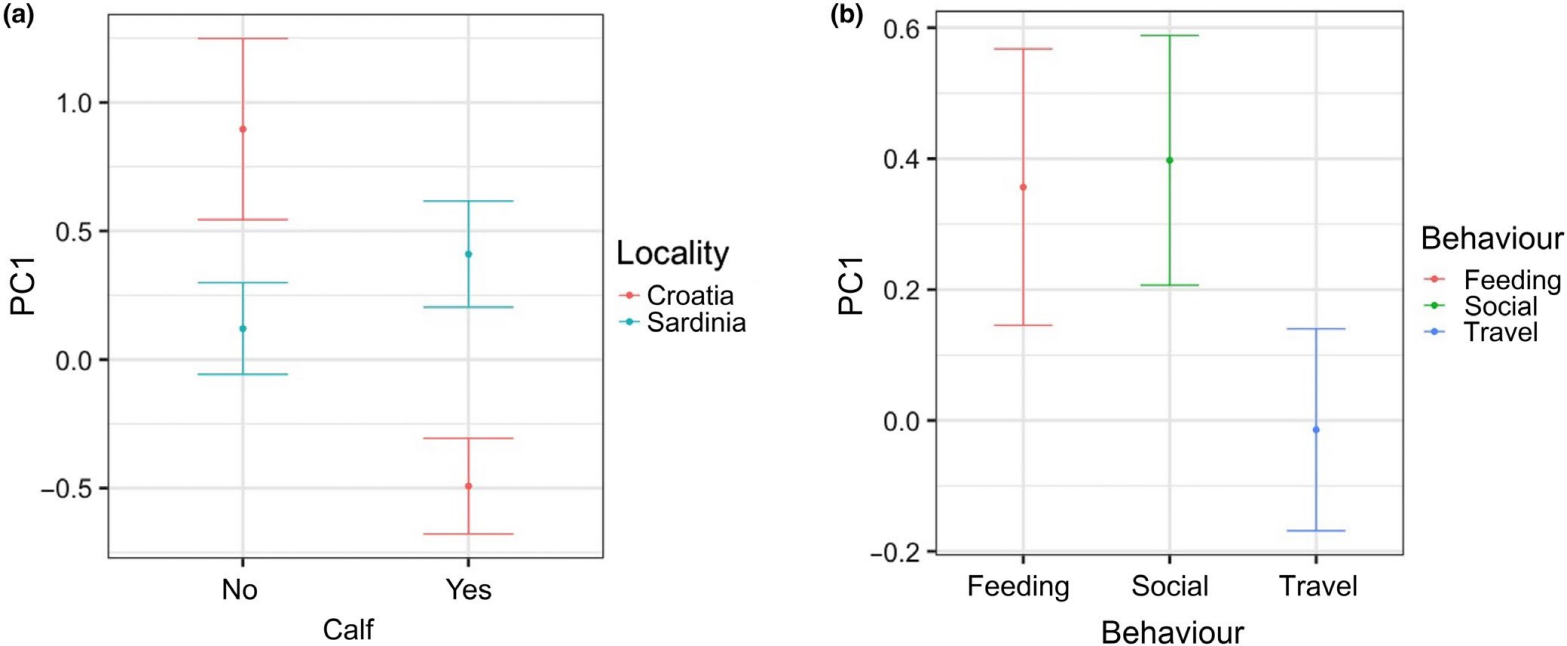
Effect of (a) the interaction between locality and calf and (b) behavior on the spectral property of the whistles (PC1) as predicted by the GLMM (elaborated with the package “nlme” in R)

**Figure 7:**
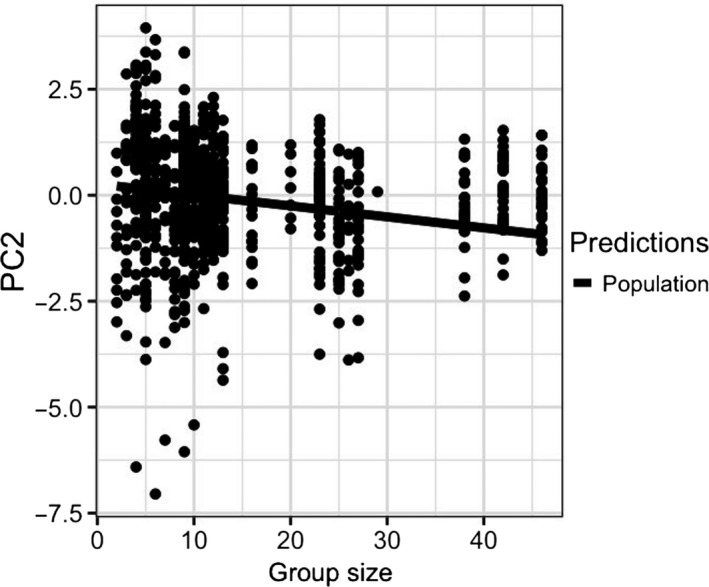
Effect of group size on the temporal property and frequency modulation of the whistles (PC2) as predicted by the GLMM (elaborated with the package “nlme” in R)

The socio‐behavioral variables, such as behavior, group size, and calf presence, have also had important influences on bottlenose dolphins’ whistle. This was highlighted by the association of PC1 with behavioral state and with the interaction between calf presence and locality (Table 8). In particular, PC1: (a) in the presence of calves, decreased in Croatia while increased in Sardinia and (b) increased during foraging and socializing compared to travel (Figure 6). Thus, min, max, start, and end frequencies (the negatively correlated characteristics to PC1) in Croatia tend to increase in the presence of calves, while they tend to decrease in Sardinia and were lower during foraging and socializing compared to travel. The PC2 was associated only with group size (Table 9) and decreased with the increasing group size (Figure 7). Thus duration, number of inflection points, and frequency range tend to increase, as the group size increases in both localities.

**Table 8:** Generalized linear mixed‐effect model (GLMM) with socio‐behavioral explanatory variables

Effect				
Fixed effects	Value	*SE*	*t*‐value	*p*‐value
(Intercept)	1.05078	0.39361	2.66960	.00780
Locality	−0.77516	0.38104	−2.03431	.04230
Calf	−1.38782	0.38210	−3.63212	.00030
Beh ‐ Social	0.04086	0.25582	0.15973	.87310
Beh ‐ Travel	−0.37084	0.21771	−1.70334	.08890
Locality: Calf	1.67713	0.46187	3.63121	.00030

Random effects	*SD* (Intercept)	Residual		
Group (Intercept)	0.823	1.462		

ANOVA between models	*df*	AIC	Likelihood ratio test	*p‐*value
Full model	12	3,046.503	−1511.252	
Best model	8	3,041.921	−1512.566	.4906

Note

The upper section shows the significant effects of the assessed explanatory variables on PC1. Value, standard errors (*SE*), *t*‐values, and significance level (*p*‐value) for variables retained in the best model are provided for fixed effects (explanatory variables), while estimates of the standard deviations (*SD*) are reported for random effects (group). The lower section presents the results of the model selection and significance of dropping the nonsignificant variables from the full model to obtain the best model. “:” = interaction.

**Table 9:** Generalized linear mixed‐effect model (GLMM) with socio‐behavioral explanatory variables

Effect				
Fixed effects	Value	*SE*	*t*‐value	*p*‐value
(Intercept)	0.26496	0.13794	1.920898	.0551
Group size	−0.02590	0.00843	−3.073734	.0022

Random effects	*SD* (Intercept)	Residual		
Group (Intercept)	0.663	1.212		

ANOVA between models	*df*	AIC	Likelihood ratio test	*p‐*value
Full model	12	2,733.438	−1354.719	
Best model	4	2,728.053	−1360.026	.2245

Note

The upper section shows the significant effects of the assessed explanatory variables on PC2. Value, standard errors (*SE*), *t*‐values, and significance level (*p*‐value) for variables retained in the best model are provided for fixed effects (explanatory variables), while estimates of the standard deviations (*SD*) are reported for random effects (group). The lower section presents the results of the model selection and significance of dropping the nonsignificant variables from the full model to obtain the best model.

## 4 DISCUSSION

### 4.1 Geographic variability

The variability in acoustic communication among dolphin populations can be affected by geographic distance and isolation, genetic differentiation, adaptation to the local acoustic and social environment, or the concomitant action of these different factors (Ey & Fischer, 2009; Parks et al., 2009; Sun et al., 2013; Wilczynski & Ryan, 1999). Based on the GLMM models run with the geographical and anthropogenic variables (noise and boat presence), only PC2 was influenced solely by locality. Thus, duration, number of inflection points, and frequency range would seem to be characteristics of the whistles influenced mainly by the specific population. This result is consistent with the fact that duration and inflection points may reflect information about the identity of the individuals (Steiner, 1981). However, this result must be taken with caution because other factors, which were not considered in the present study, could have affected the whistle characteristics expressed as PC2. Moreover, as we have recorded some of the photo‐identified individuals several times, in both locality, this could have influenced our finding, despite the analytical methods adopted to contain this aspect.

The geographically distant and genetically distinct populations may exhibit acoustic variability (Funk et al., 2005; Irwin et al., 2008). Although genetic characterization of the dolphins inhabiting the two study areas is lacking, the acoustic variability here (see also La Manna et al., 2017) may be consistent with previous studies on the genetic structure of bottlenose dolphin in the Mediterranean. Indeed, Gaspari et al. (2015) have found that the genetic structure of the main population groups of bottlenose dolphin corresponds to the main Basin of the Mediterranean (Tyrrhenian, Ionian, Adriatic, Aegean, and Levantine seas), suggesting reproductive isolation at this scale (Delarue et al., 2009). Therefore, even if the gene flow among distant areas of the Mediterranean Sea is still mediated by individuals of the pelagic ecotype (Gaspari et al., 2015; Natoli, Birkun, Aguilar, Lopez, & Rus Hoelzel, 2005), the absence of a constant contact between the Croatia and Sardinia populations (located 2000 km apart) can be assumed. Consequently, the geographical variability of some characteristics of their whistle can be inferred (Hoffmann et al., 2012). However, where acoustic communication is mediated by learning processes (as in cetaceans), and therefore by cultural transmission (Bain, 1986; Ford, 1991; Janik & Slater, 2000), populations that are geographically but not genetically distant may also manifest acoustic variability (Camargo, Rollo, Giampaoli, & Bellini, 2007; Rendell & Whitehead, 2005; Rossi‐Santos & Podos, 2006). Regardless of the mechanism responsible for such variability whether geographic distance and isolation, genetic variability, and/or cultural drift (Wilkins et al., 2012), our results provide evidence for variability in whistles between the two populations.

### 4.2 Influence of noise and boat presence on whistle structure

The variation in acoustic communication can manifest itself as an adaptation to local noise and boat presence conditions, even in the absence of geographic isolation, genetic causes, or cultural drift (Ansmann et al., 2007; Baron et al., 2008; van Ginkel et al., 2017; La Manna et al., 2013; Leao et al., 2016; Luís, Couchinho, & Santos, 2014; Morisaka et al., 2005; Papale et al., 2015; Rako‐Gospić & Picciulin, 2016). According to the acoustic adaptation hypothesis, the signals emitted by an animal are adapted to the environment in which it lives, to minimize degradation, maximize signal transmission, and ensure long‐range communication (Ey & Fischer, 2009). Moreover, sound transmission in the ocean can change as a function of pressure, temperature, salinity, depth, bathymetric contour, among a range of other factors, and thus varies geographically and over time (Richardson, Greene, Malme, & Thomson, 1995). The soundscape within an environment is the result of the different sources of sound produced by natural conditions, living organisms, and human activities. The SAN levels described in this study were indicative of two different acoustic environments characterizing the two areas during the periods recorded, considering both the low and mid octave bands (125 Hz and 20 kHz). The GLMM models have associated the spectral property of the whistles (PC1) to the lowest frequency SAN level (125 Hz band, and the correlated bands at 500 Hz and 1,000 Hz) mainly in Croatia. The bottlenose dolphin produces whistles with energy mostly between 2 and 20 kHz (Richardson et al., 1995). However, the lowest frequencies of the whistles produced by the dolphins in both study areas were lower, and dolphins’ ability to hear sound at 100 Hz and to produce sounds at 200 Hz is known (Herzing, 1996; Johnson, 1968; Turl, 1993). Even if with our approach we cannot prove causation, other studies found a similar association between low‐frequency noise and whistle characteristics. For example, Marley et al. (2017) found that noise in the 1 kHz octave band was more strongly associated with dolphin whistle characteristics than noise in the 16 kHz and 32 kHz octave bands. Moreover, the association between low‐frequency noise and the spectral property of the whistles (min, max, start, and end frequencies) found mainly in Croatia is consistent with similar frequency shift related to low‐frequency noise in other studies (Fouda et al., 2018; van Ginkel et al., 2017; Luís et al., 2014; Marley et al., 2017; May‐Collado & Wartzok, 2008; Rako‐Gospić & Picciulin, 2016; Wang et al., 1995). The frequency shift is recognized as one among the several mechanisms that dolphins use to cope with noise, likely aimed at increasing transmission efficiency and detectability of their acoustic signals (Rako‐Gospić & Picciulin, 2016). Another mechanism that could explain the increased whistle frequencies is the Lombard effect, where the vocal production changes in response to an increase in amplitude of background noise (Brumm & Zollinger, 2011). Even though in Sardinia average levels of noise (recorded at times that whistles were recorded) were higher than in Croatia, whistle changes, in terms of shifting frequencies, were not significant compared to Croatia dolphins. A different response between the two areas was also found due to the presence of boats: Sardinian dolphins produced whistles at higher frequencies in the presence of boats compared to Croatian dolphins. The different response to noise of different populations is an evidence of the plasticity of bottlenose dolphin whistles (May‐Collado & Wartzok, 2008). These differences may be related to the diverse boat traffic and SAN levels to which the two dolphin communities are exposed, but also to other factors such as the environmental conditions (e.g., physical and chemical conditions of the water column and seabed which influence sound propagation) and natural and anthropogenic pressures (e.g., prey availability, fishing, tourism) of the two areas. At the end, dolphin's responses to such pressures, which in turn depend on the physiological state of the individuals, may influence their capacity to tolerate or habituate to disturbances.

### 4.3 Socio and behavioral influence

Whistle characteristics were influenced by the behavioral states and calf presence (PC1 only) and by group size (PC2). The influence of the behavioral context, social and foraging, on the whistle structure was independent of the population. Accordingly, a similar frequency shift in whistles was detected during the behavioral states characterized by a high level of arousal (Acevedo‐Gutièrrez & Stienessen, 2004; Hawkins, 2010; King & Janik, 2015; La Manna et al., 2013; May‐Collado & Quiñones‐Lebrón, 2014; Rako‐Gospić & Picciulin, 2016). Moreover, duration, number of inflection points, and frequency range decreased with the increasing group size, independently of the population (PC2), even if data for group size between 25 and 35 individuals were lacking. This result is consistent with the hypothesis that longer and more complex tonal sound may help group cohesion in social groups (May‐Collado et al., 2007). The calf presence shaped the whistles of Sardinia and Croatia in a different way. Many different factors, such as the number and age of the calves present, or the effect of their whistles on their mothers’ whistles, and the attempt of the calves to replicate the adult whistles can be related to such difference. These aspects need further investigation to be disentangled. Therefore, this study supports the importance of considering the social and behavioral context of the animals when investigating the acoustic structure of communication signals (Guerra, Dawson, Brough, & Rayment, 2014; Heiler et al., 2016; Marley et al., 2017), and its variability between populations to avoid misleading conclusions.

The results of the study should be interpreted considering some methodological limitations. The approach used does not allow identifying the emitter of the whistle; therefore, the answer found must be generically attributed to the group. Therefore, it is not possible to highlight the individual contribution of dolphins reactions to the presence of boats or to noise levels, and on the other hand, the response of same individuals may be oversampled. Furthermore, only some of the potential factors associated with acoustic variability were considered in the study. Other important sources of variability, either related to characteristics of vessel traffic (in terms of boat speed, behavior, and exact distance to dolphins) or to the environment such as features affecting sound propagation (i.e., water temperature, depth, and type of substrate), have not been considered here and deserve attention in future studies.

## 5 CONCLUSION

Geographic variation of the bottlenose dolphin whistles has been found in several oceans worldwide (Azevedo et al., 2007; La Manna et al., 2017; May‐Collado & Wartzok, 2008; Morisaka et al., 2005; Papale et al., 2014; Wang et al., 1995), including the Mediterranean Sea, but to our knowledge, this is the first attempt to identify the factors associated with such variability. The results provide evidence supporting the hypothesis that the geographically isolated populations of Sardinia and Croatia may have developed their communication, in response to both the acoustic environment and vessel traffic condition in which they live, and to other factors, such as cultural/genetic drift.

The anthropogenic noise in the marine environment is generally increasing (Hildebrand, 2009), and it can have a wide range of impacts on wildlife, including individual behavior, physiology (Shannon, McKenna, Angeloni, & Wittemyer, 2016), and metabolic cost (Holt, Noren, Dunkin, & Williams, 2015), with unknown consequences in the long term and at the population level. While our results are not conclusive, the variability identified in this study either provides evidence of isolated populations or supports for changes in acoustic behavior in response to differing environments, in which human activities may be among the cause of such difference. Additional research is required to improve our understanding of the causes of acoustic variability in dolphin populations and to improve management of anthropogenic noise and boat traffic in both areas.

## CONFLICT OF INTEREST

None Declared.

## AUTHORS’ CONTRIBUTION

Gabriella La Manna, Nikolina Rako‐Gospic, Giulia Ceccherelli, Silvia Bonizzoni, and Gianluca Sarà conceived and designed the experiments. Gabriella La Manna, Nikolina Rako‐Gospic, and Federica Gatti performed the experiments. Gabriella La Manna, Nikolina Rako‐Gospic, and Federica Gatti analyzed the data. Gabriella La Manna, Nikolina Rako‐Gospic, Gianluca Sarà, Silvia Bonizzoni, and Giulia Ceccherelli wrote the paper.

## Data Availability

The dataset used in the analysis can be found on Dryad https://doi.org/10.5061/dryad.1jwstqjr3
