# Supplementary material for: Whistle variation in Mediterranean common bottlenose dolphin: The role of geographical, anthropogenic, social, and behavioral factors
Source: Ecol Evol. 2020 Feb 5;10(4):1971–87. doi: 10.1002/ece3.6029 (PMC7042681; doi:10.1002/ece3.6029)

Electronic supplementary material

ES1. Generalised linear mixed-effect model selection step-by-step. In each model table, the upper section shows the effects of the assessed explanatory anthropogenic variables on the first principal component (PC1). Value, standard errors (SE), t-values, and significance level (*P*-value) are provided for fixed effects (explanatory variables), whilst estimates of the standard deviations (SD) are reported for random effects (group). Variables dropped in each subsequent model are given in bold. The lower section of each of the models where a variable has been dropped presents the significance of dropping each variable from the previous model. Akaike's Information Criterion (AIC) is also provided for each model. Further, the anova between the full model and the best model is also provided at the end of the section.

Full model

AIC: 3037.322

EFFECT				
Fixed effects	**Value**	**SE**	**t-value**	***P*-value**
(Intercept)	1.97202	0.97139	2.03012	0.04270
Locality	-0.96351	1.58427	-0.60817	0.54330
SPL 125 Hz	-0.02570	0.01249	-2.05824	0.03990
SPL 2000 Hz	-0.00307	0.01316	-0.23360	0.81540
SPL 20000 Hz	0.00251	0.00976	0.25661	0.79760
Boat	0.26513	0.22567	1.17485	0.24040
Locality : SPL 125 Hz	0.03539	0.01819	1.94581	0.05210
Locality : SPL 2000 Hz	-0.00886	0.02000	-0.44286	0.65800
**Locality : SPL 20000 Hz**	**-0.00233**	**0.01626**	**-0.14350**	**0.88590**
Locality : Boat	-1.12258	0.30523	-3.67784	0.00030
Random effects	**SD** (Intercept)	Residual		
Group (Intercept)	0.7356	1.4541		

1st drop: SPL 20000 Hz : Locality

EFFECT				
Fixed effects	**Value**	**SE**	**t-value**	***P*-value**
(Intercept)	2.00426	0.94160	2.12856	0.0336
Locality	-1.08738	1.31706	-0.82562	0.4093
SPL 125 Hz	-0.02567	0.01248	-2.05693	0.0400
SPL 2000 Hz	-0.00268	0.01286	-0.20808	0.8352
**SPL 20000 Hz**	**0.00166**	**0.00778**	**0.21367**	**0.8309**
Boat	0.26291	0.22470	1.17002	0.2424
SPL 125 Hz : Locality	0.03534	0.01818	1.94463	0.0522
SPL 2000 Hz : Locality	-0.00984	0.01879	-0.52374	0.6006
Boat : Locality	-1.12410	0.30490	-3.68680	0.0002
Random effects	**SD** (Intercept)	Residual		
Group (Intercept)	0.7343	1.4543		
**ANOVA between models**				
**Term dropped**	**df**	**AIC**	**Likelihood ratio test**	***P*-value**
Full model	12	3037.322	-1506.661	
1st drop model	11	3035.343	-1506.671	0.8857

2nd drop: SPL 20000

EFFECT				
Fixed effects	**Value**	**SE**	**t-value**	***P*-value**
(Intercept)	2.07368	0.88442	2.34469	0.0193
Locality	-1.06196	1.31057	-0.81030	0.4180
SPL 125 Hz	-0.02558	0.01246	-2.05213	0.0405
SPL 2000 Hz	-0.00195	0.01240	-0.15763	0.8748
Boat	0.25817	0.22359	1.15469	0.2486
SPL 125 Hz : Locality	0.03526	0.01816	1.94145	0.0526
**SPL 2000 Hz : Locality**	**-0.00982**	**0.01878**	**-0.52298**	**0.6011**
Boat : Locality	-1.11519	0.30204	-3.69218	0.0002
Random effects	**SD** (Intercept)	Residual		
Group	0.7349	1.4543		
**ANOVA between models**				
**Term dropped**	**df**	**AIC**	**Likelihood ratio test**	***P*-value**
1st drop model	11	3035.343	-1506.671	
2nd drop model	10	3033.389	-1506.694	0.8299

3rd Drop: SPL 2000 Hz : Locality

EFFECT				
Fixed effects	**Value**	**SE**	**t-value**	***P*-value**
(Intercept)	2.2102469	0.84627	2.61175	0.00920
Locality	-1.3800683	1.16408	-1.18554	0.23620
SPL 125 Hz	-0.022339	0.01079	-2.06950	0.03880
**SPL 2000 Hz**	**-0.0062356**	**0.00932**	**-0.66888**	**0.50380**
Boat	0.2522307	0.22335	1.12932	0.25910
SPL 125 Hz : Locality	0.0282758	0.01224	2.31062	0.02110
Boat : Locality	-1.1230398	0.30156	-3.72408	0.00020
Random effects	**SD** (Intercept)	Residual		
Group (Intercept)	0.7373	1.454		

ANOVA between models

**Term dropped**	**df**	**AIC**	**Likelihood ratio test**	***P*-value**
2nd drop model	10	3033.389	-1506.694	
3rd drop model	9	3031.664	-1506.832	0.5996

4th drop: SPL 2000 Hz

EFFECT				
Fixed effects	**Value**	**SE**	**t-value**	***P*-value**
(Intercept)	2.00274	0.78787	2.54196	0.0112
Locality	-1.38636	1.16338	-1.19167	0.2338
SPL 125 Hz	-0.02691	0.00834	-3.22695	0.0013
Boat	0.24922	0.22314	1.11686	0.2644
SPL 125 Hz : Locality	0.02874	0.01221	2.35439	0.0188
Boat : Locality	-1.13783	0.30063	-3.78484	0.0002
Random effects	**SD** (Intercept)	Residual		
Group (Intercept)	0.7356	1.4549		
**ANOVA between models**				
**Term dropped**	**df**	**AIC**	**Likelihood ratio test**	***P*-value**
3rd drop model	9	3031.664	-1506.832	
4th drop model	8	3030.115	-1507.057	0.502

Anova between the full model and the best model

ANOVA between models				
**Term dropped**	**df**	**AIC**	**Likelihood ratio test**	***P*-value**
None (Full model)	12	3037.322	-1506.661	
4th drop model	8	3030.115	-1507.057	0.9393

Model Validation

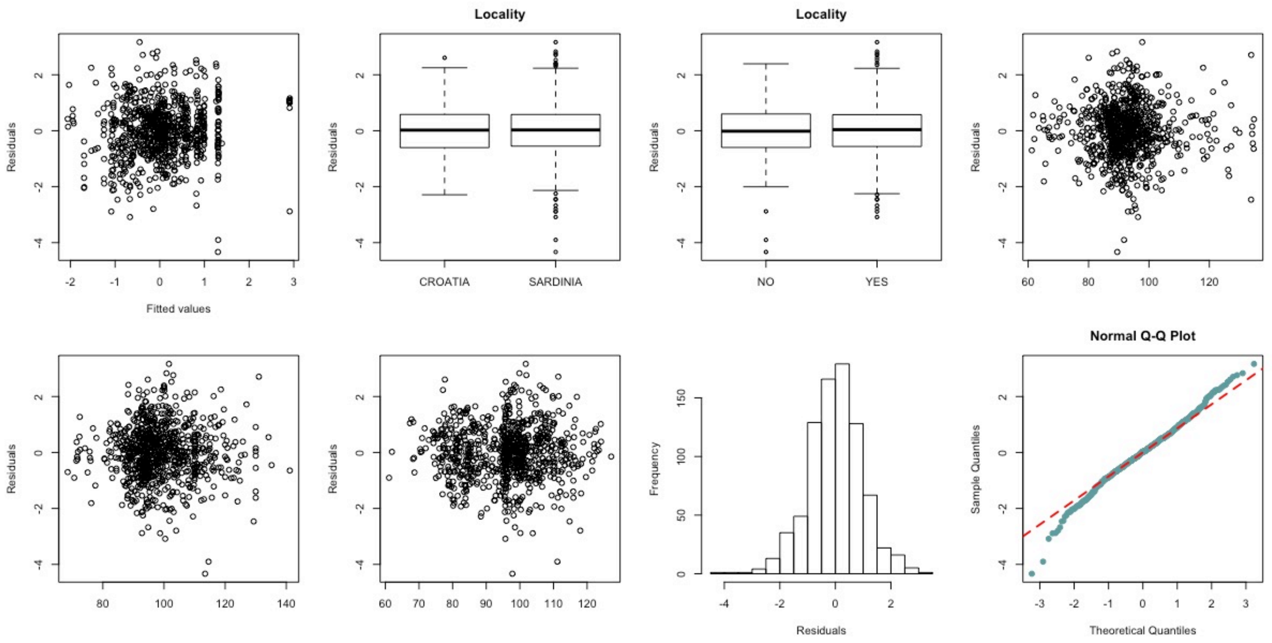

ES2. Generalised linear mixed-effect model selection step-by-step. In each model table, the upper section shows the effects of the assessed explanatory anthropogenic variables on the second principal component (PC2). Value, standard errors (SE), t-values, and significance level (*P*-value) are provided for fixed effects (explanatory variables), whilst estimates of the standard deviations (SD) are reported for random effects (group). Variables dropped in each subsequent model are given in bold. The lower section of each of the models where a variable has been dropped presents the significance of dropping each variable from the previous model. Akaike's Information Criterion (AIC) is also provided for each model. Further, the anova between the full model and the best model is also provided at the end of the section.

Full model

AIC: 2741.467

EFFECT<br>***Fixed effects***	**Value**	**SE**	**t-value**	***P*-value**
(Intercept)	0.34272	0.81924	0.41834	0.6758
Locality	-0.18678	1.33104	-0.14032	0.8884
SPL 125 Hz	-0.00013	0.01041	-0.01274	0.9898
SPL 2000 Hz	0.00279	0.01098	0.25361	0.7999
SPL 20000 Hz	-0.00962	0.00812	-1.18398	0.2368
Boat	-0.06043	0.19058	-0.31710	0.7513
SPL 125 Hz : Locality	-0.00546	0.01517	-0.35969	0.7192
SPL 2000 Hz : Locality	0.01110	0.01670	0.66454	0.5066
**SPL 20000 Hz : Locality**	**-0.00041**	**0.01355**	**-0.03030**	**0.9758**
Boat : Locality	0.30196	0.25849	1.16819	0.2431
Random effects	**SD**<br>(Intercept)	Residual		
Group	0.6867	1.2052		

1st drop: SPL 20000 Hz : Locality

EFFECT				
Fixed effects	**Value**	**SE**	**t-value**	***P*-value**
(Intercept)	0.34868	0.79516	0.43850	0.6612
Locality	-0.20919	1.11024	-0.18842	0.8506
SPL 125 Hz	-0.00013	0.01040	-0.01232	0.9902
SPL 2000 Hz	0.00285	0.01074	0.26565	0.7906
SPL 20000 Hz	-0.00977	0.00648	-1.50770	0.1321
Boat	-0.06100	0.18979	-0.32142	0.7480
**SPL 125 Hz : Locality**	**-0.00547**	**0.01516**	**-0.36045**	**0.7186**
SPL 2000 Hz : Locality	0.01093	0.01572	0.69542	0.4870
Boat : Locality	0.30192	0.25828	1.16897	0.2428
Random effects	**SD** (Intercept)	Residual		
Group	0.6869	1.20526		
**ANOVA between models**				
**Term dropped**	**df**	**AIC**	**Likelihood ratio test**	***P*-value**
Full model	12	2741.467	-1358.734	
1st drop model	11	2791.360	-1358.734	0.9757

2nd drop: SPL 125 Hz:Locality

EFFECT				
Fixed effects	**Value**	**SE**	**t-value**	***P*-value**
(Intercept)	0.39211	0.78543	0.49923	0.6178
Locality	-0.30773	1.07498	-0.28627	0.7748
**SPL 125 Hz**	**-0.00273**	**0.00746**	**-0.36641**	**0.7142**
SPL 2000 Hz	0.00475	0.00936	0.50724	0.6121
SPL 20000 Hz	-0.00971	0.00647	-1.50009	0.1340
Boat	-0.06103	0.18967	-0.32176	0.7477
SPL 2000 Hz : Locality	0.00676	0.01065	0.63455	0.5259
Boat : Locality	0.30116	0.25811	1.16681	0.2437
Random effects	**SD** (Intercept)	Residual		
Group	0.6863	1.2054		
**ANOVA between models**				
**Term dropped**	**df**	**AIC**	**Likelihood ratio test**	***P*-value**
1st drop model	11	2739.468	-1358.734	
2nd drop model	10	2737.599	-1358.800	0.7171

3rd Drop: SPL 125 Hz

EFFECT<br>***Fixed effects***	**Value**	**SE**	**t-value**	***P*-value**
(Intercept)	0.34776	0.77539	0.44850	0.654
Locality	-0.30871	1.07394	-0.28745	0.774
SPL 2000 Hz	0.00278	0.00764	0.36364	0.716
SPL 20000 Hz	-0.00978	0.00647	-1.51170	0.131
Boat	-0.05778	0.18940	-0.30508	0.760
**SPL 2000 Hz : Locality**	**0.00660**	**0.01064**	**0.62042**	**0.535**
Boat : Locality	0.29942	0.25782	1.16138	0.246
Random effects	**SD**<br>(Intercept)	Residual		
Group	0.6832	1.2058		
**ANOVA between models**				
**Term dropped**	**df**	**AIC**	**Likelihood ratio test**	***P*-value**
2nd drop model	10	2737.599	-1358.800	
3rd drop model	9	2735.734	-1358.867	0.714

4th drop: SPL 2000 Hz:Locality

EFFECT				
Fixed effects	**Value**	**SE**	**t-value**	***P*-value**
(Intercept)	0.06951	0.62912	0.11048	0.9121
Locality	0.34255	0.22125	1.54826	0.1220
**SPL 2000 Hz**	**0.00557**	**0.00616**	**0.90528**	**0.3656**
SPL 20000 Hz	-0.00985	0.00647	-1.52422	0.1279
Boat	-0.04659	0.18859	-0.24704	0.8049
Boat : Locality	0.30871	0.25662	1.20298	0.2294
Random effects	**SD** (Intercept)	Residual		
Group	0.6774	1.2068		
**ANOVA between models**				
**Term dropped**	**df**	**AIC**	**Likelihood ratio test**	***P*-value**
3rd drop model	9	2735.734	-1358.867	
4th drop model	8	2734.119	-1359.059	0.5351

5th drop:SPL 2000 Hz

EFFECT				
Fixed effects	**Value**	**SE**	**t-value**	***P*-value**
(Intercept)	0.36764	0.53628	0.6855	0.49320
Locality	-0.00701	0.00565	-1.2408	0.21510
SPL 20000 Hz	-0.03832	0.18833	-0.2035	0.83880
Boat	0.29414	0.21464	1.3704	0.17100
**Boat : Locality**	**0.31348**	**0.25638**	**1.2227**	**0.22180**
Random effects	**SD** (Intercept)	Residual		
Group	0.6754	1.2076		
**ANOVA between models**				
**Term dropped**	**df**	**AIC**	**Likelihood ratio test**	***P*-value**
4th drop model	8	2734.119	-1359.059	
5rd drop model	7	2732.943	-1359.472	0.3638

6th drop: Boat : Locality

EFFECT				
Fixed effects	**Value**	**SE**	**t-value**	***P*-value**
(Intercept)	0.17786	0.50909	0.34936	0.72690
Locality	-0.00593	0.00557	-1.06519	0.28710
**SPL 20000 Hz**	**0.13493**	**0.12442**	**1.08440**	**0.27850**
Boat	0.45295	0.16339	2.77223	0.00570
Random effects	**SD** (Intercept)	Residual		
Group	0.6519	1.2113		
**ANOVA between models**				
**Term dropped**	**df**	**AIC**	**Likelihood ratio test**	***P*-value**
5th drop model	7	2732.943	-1359.472	
6th drop model	6	2732.385	-1360.192	0.2299

7th drop: SPL 20000 Hz

EFFECT				
Fixed effects	**Value**	**SE**	**t-value**	***P*-value**
(Intercept)	-0.3419054	0.1510043	-2.26421	0.02380
Locality	0.3696547	0.142512	2.59385	0.00970
**Boat**	**0.12691**	**0.12436**	**1.02055**	**0.30780**
Random effects	**SD** (Intercept)	Residual		
Group	0.6574	1.2116		
**ANOVA between models**				
**Term dropped**	**df**	**AIC**	**Likelihood ratio test**	***P*-value**
6th drop model	6	2732.385	-1360.192	
7th drop model	5	2731.521	-1360.761	0.2864

8th drop: Boat

EFFECT				
Fixed effects	**Value**	**SE**	**t-value**	***P*-value**
(Intercept)	-0.2669942	0.1314596	-2.03100	0.04260
Locality	0.3762232	0.1427245	2.63601	0.00860
Random effects	**SD** (Intercept)	Residual		
Group				
**ANOVA between models**				
**Term dropped**	**df**	**AIC**	**Likelihood ratio test**	***P*-value**
7th drop model	6	2731.521	-1360.761	
8th drop model	5	2730.563	-1361.282	0.3074

Anova between the full model and the best model

ANOVA between models				
**Term dropped**	**df**	**AIC**	**Likelihood ratio test**	***P*-value**
<None> (Full model)	12	2741.467	-1358.734	
8th drop model	4	2730.563	-1361.282	0.7473

Model Validation

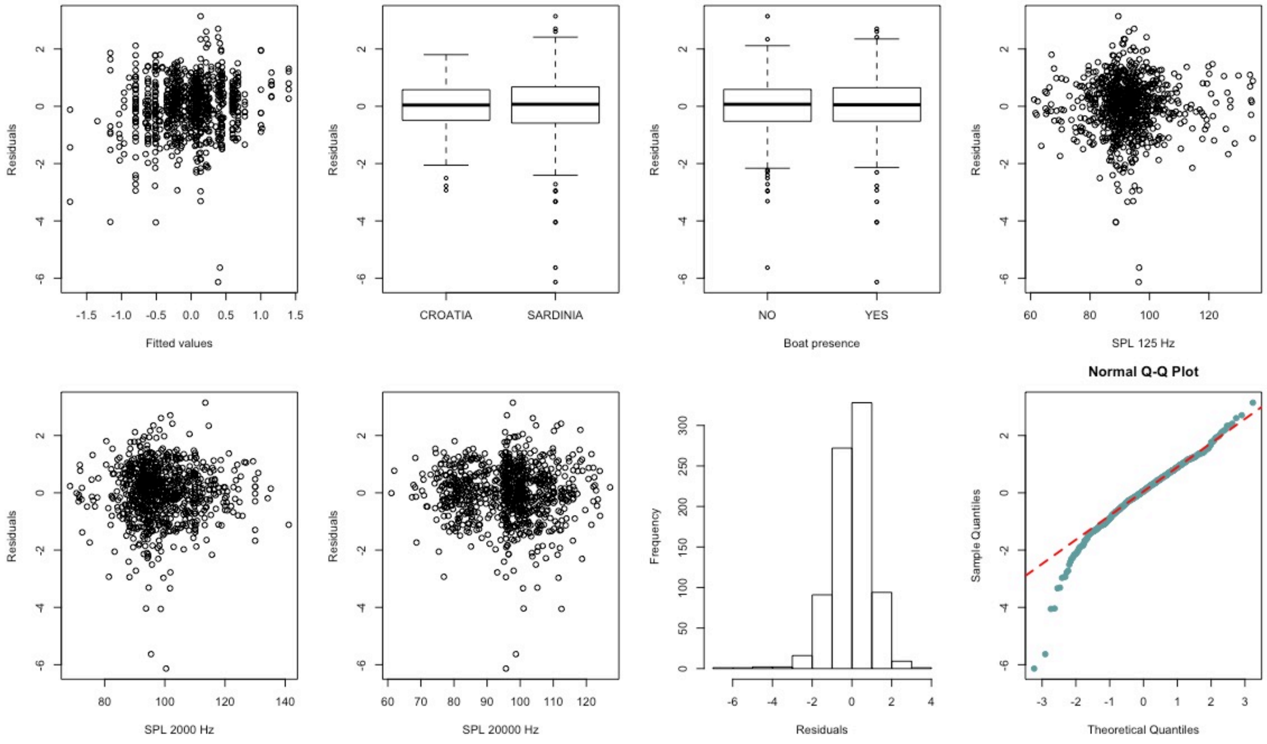

ES3. Generalised linear mixed-effect model selection step-by-step. In each model table, the upper section shows the effects of the assessed explanatory socio-behavioural variables on the first principal component (PC1). Value, standard errors (SE), t-values, and significance level (*P*-value) are provided for fixed effects (explanatory variables), whilst estimates of the standard deviations (SD) are reported for random effects (group). Variables dropped in each subsequent model are given in bold. The lower section of each of the models where a variable has been dropped presents the significance of dropping each variable from the previous model. Akaike's Information Criterion (AIC) is also provided for each model. Further, the anova between the full model and the best model is also provided at the end of the section.

Full model

AIC: 3046.503

EFFECT<br>***Fixed effects***	**Value**	**SE**	**t-value**	***P*-value**
(Intercept)	0.81723	0.44572	1.833513	0.0671
Locality	-0.62575	0.58413	-1.071251	0.2844
Group size	0.00318	0.01672	0.190299	0.8491
Calf	-1.38897	0.42403	-3.275671	0.0011
Beh - Social	0.08945	0.52736	0.169621	0.8654
Beh - Travel	-0.11066	0.35618	-0.310683	0.7561
Locality : Group size	0.04530	0.04444	1.019509	0.3083
Locality : Calf	1.55707	0.50634	3.075153	0.0022
Locality : Beh - Travel	-0.30072	0.61664	-0.487678	0.6259
Locality : Beh - Social	-0.54511	0.47031	-1.159038	0.2468
Random effects	**SD**<br>(Intercept)	Residual		
Group (Intercept)	0.7712	1.4588		

1st drop: Beh : Locality

EFFECT				
Fixed effects	**Value**	**SE**	**t-value**	***P*-value**
(Intercept)	0.98896	0.39446	2.50713	0.0124
Locality	-0.96874	0.46733	-2.07292	0.0385
Group size	0.01069	0.01528	0.69936	0.4845
Calf	-1.48908	0.40913	-3.63959	0.0003
Beh - Social	-0.06794	0.25735	-0.26399	0.7919
Beh - Travel	-0.43396	0.22714	-1.91056	0.0564
**Locality : Group size**	**0.03981**	**0.04376**	**0.90989**	**0.3632**
Locality : Calf	1.66985	0.48473	3.44491	0.0006
Random effects	**SD** (Intercept)	Residual		
Group	0.7589	1.4615		
**ANOVA between models**				
**Term dropped**	**df**	**AIC**	**Likelihood ratio test**	***P*-value**
Full model	12	3046.503	-1511.252	
1st drop model	10	3043.870	-1511.935	0.5049

2nd drop: Group size : Locality

EFFECT				
Fixed effects	**Value**	**SE**	**t-value**	***P*-value**
(Intercept)	0.95142	0.39309	2.42035	0.0157
Locality	-0.71585	0.37429	-1.91253	0.0562
Group size	0.01595	0.01414	1.12814	0.2596
Calf	-1.53537	0.40716	-3.77094	0.0002
Beh - Social	-0.04375	0.25690	-0.17029	0.8648
Beh - Travel	-0.46168	0.22513	-2.05075	0.0406
Locality : Calf	1.78197	0.47119	3.78187	0.0002
Random effects	**SD** (Intercept)	Residual		
Group	0.7653	1.4615		
**ANOVA between models**				
**Term dropped**	**df**	**AIC**	**Likelihood ratio test**	***P*-value**
1st drop model	10	3043.870	-1511.935	
2nd drop model	9	3042.702	-1512.351	0.3616

3rd drop: Group size

EFFECT				
Fixed effects	**Value**	**SE**	**t-value**	***P*-value**
(Intercept)	1.04525	0.38916	2.68591	0.00740
Locality	-0.76319	0.37680	-2.02547	0.04320
Calf	-1.37031	0.37804	-3.62482	0.00030
Beh - Social	0.03105	0.25288	0.12279	0.90230
Beh - Travel	-0.37678	0.21570	-1.74681	0.08110
Locality : Calf	1.65110	0.45612	3.61986	0.00030
Random effects	**SD** (Intercept)	Residual		
Group	0.7913	1.4598		
**ANOVA between models**				
**Term dropped**	**df**	**AIC**	**Likelihood ratio test**	***P*-value**
2nd drop model	9	3042.702	-1512.351	
3rd drop model	8	3041.921	-1512.960	0.2698

Anova between the full model and the best model

ANOVA between models				
**Term dropped**	**df**	**AIC**	**Likelihood ratio test**	***P*-value**
<None> (Full model)	12	3046.503	-1511.252	
3rd drop model	8	3041.921	-1512.566	0.4906

Model Validation

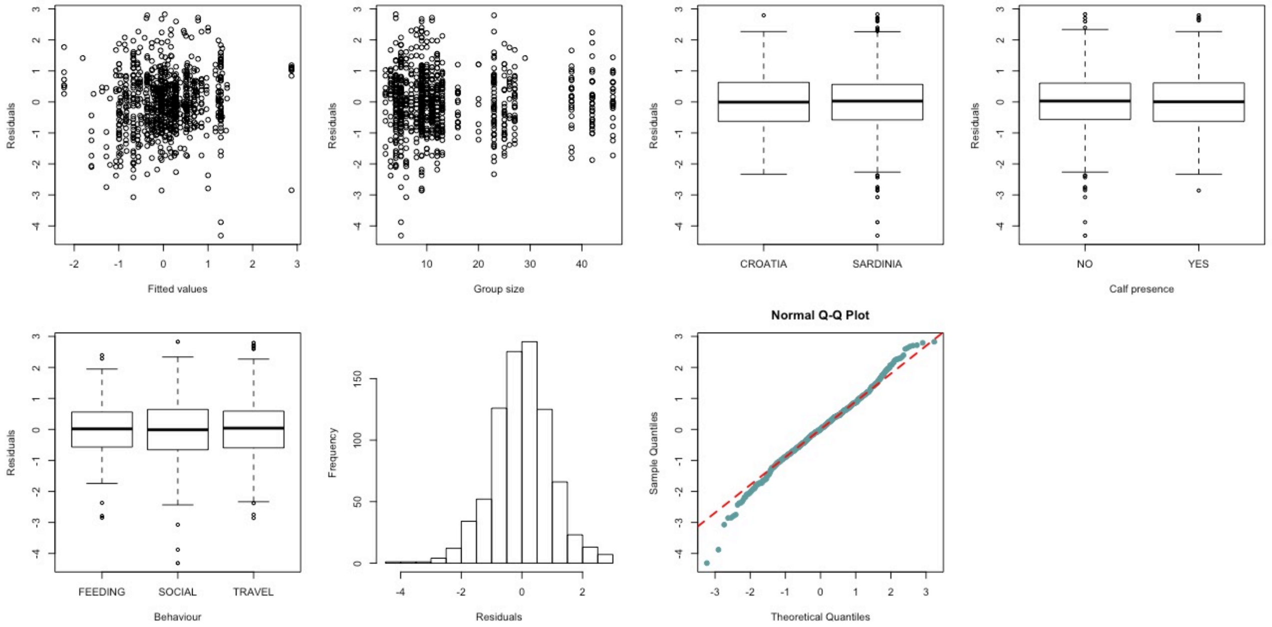

ES4. Generalised linear mixed-effect model selection step-by-step. In each model table, the upper section shows the effects of the assessed explanatory socio-behavioural variables on the second principal component (PC2). Value, standard errors (SE), t-values, and significance level (*P*-value) are provided for fixed effects (explanatory variables), whilst estimates of the standard deviations (SD) are reported for random effects (group). Variables dropped in each subsequent model are given in bold. The lower section of each of the models where a variable has been dropped presents the significance of dropping each variable from the previous model. Akaike's Information Criterion (AIC) is also provided for each model. Further, the anova between the full model and the best model is also provided at the end of the section.

Full model

AIC: 2733.438

EFFECT *Fixed effects*	Value	SE	t-value	*P*-value
(Intercept)	0.33624	0.36717	0.91577	0.3601
Locality	0.34134	0.48110	0.70949	0.4782
Group size	0.00662	0.01376	0.48103	0.6306
Calf	-0.51490	0.34935	-1.47390	0.1409
Beh - Social	0.18792	0.43389	0.43311	0.6651
Beh - Travel	-0.50581	0.29364	-1.72252	0.0854
Locality : Group size	-0.06794	0.03656	-1.85819	0.0635
**Locality : Calf**	**0.50904**	**0.41700**	**1.22072**	**0.2226**
Locality : Beh - Travel	-0.42921	0.50751	-0.84573	0.3980
Locality : Beh - Social	0.38875	0.38767	1.00280	0.3163
Random effects	**SD** (Intercept)	Residual		
Group (Intercept)	0.6320	1.2049		

1st drop: Calf : Locality

EFFECT				
Fixed effects	**Value**	**SE**	**t-value**	***P*-value**
(Intercept)	0.06114	0.28713	0.2129	0.8314
Locality	0.63618	0.40876	1.5563	0.1201
Group size	0.00055	0.01263	0.0436	0.9652
**Calf**	**-0.15214**	**0.18595**	**-0.8182**	**0.4135**
Beh - Social	0.32025	0.41594	0.7699	0.4416
Beh - Travel	-0.38521	0.27532	-1.3991	0.1622
Locality : Group size	-0.05653	0.03487	-1.6212	0.1054
Locality : Beh - Travel	-0.57327	0.48714	-1.1768	0.2396
Locality : Beh - Social	0.29043	0.37576	0.7729	0.4398
Random effects	**SD** (Intercept)	Residual		
Group	0.6175	1.2078		
**ANOVA between models**				
**Term dropped**	**df**	**AIC**	**Likelihood ratio test**	***P*-value**
Full model	12	2733.438	-1354.719	
1st drop model	11	2732.925	-1355.46	0.2227

2nd drop: Calf

EFFECT				
Fixed effects	**Value**	**SE**	**t-value**	***P*-value**
(Intercept)	-0.02573	0.26560	-0.0968739	0.9229
Locality	0.69783	0.39979	1.7455008	0.0813
Group size	-0.00175	0.01224	-0.143102	0.8862
Beh - Social	0.34238	0.41339	0.8282222	0.4078
Beh - Travel	-0.36876	0.27413	-1.3452246	0.179
Locality : Group size	-0.06107	0.03432	-1.7795917	0.0756
**Locality : Beh - Travel**	**-0.56596**	**0.48521**	**-1.166419**	**0.2438**
**Locality : Beh - Social**	**0.27280**	**0.37418**	**0.7290663**	**0.4662**
Random effects	**SD** (Intercept)	Residual		
Group	0.6128	1.2088		
**ANOVA between models**				
**Term dropped**	**df**	**AIC**	**Likelihood ratio test**	***P*-value**
1st drop model	11	2732.925	-1355.463	
2nd drop model	10	2731.599	-1355.800	0.4115

3rd drop: Beh : Locality

EFFECT				
Fixed effects	**Value**	**SE**	**t-value**	***P*-value**
(Intercept)	0.04169	0.24883	0.1675214	0.867
Locality	-0.00950	0.01176	-0.8075576	0.4196
Group size	0.70536	0.34010	2.0739774	0.0384
**Beh - Social**	**-0.17989**	**0.21133**	**-0.8512257**	**0.3949**
**Beh - Travel**	**-0.19798**	**0.18189**	**-1.0884332**	**0.2768**
Locality : Group size	-0.05810	0.03523	-1.6492696	0.0995
Random effects	**SD** (Intercept)	Residual		
Group	0.6523	1.2072		
**ANOVA between models**				
**Term dropped**	**df**	**AIC**	**Likelihood ratio test**	***P*-value**
2nd drop model	10	2731.599	-1355.80	
3rd drop model	8	2731.220	-1357.61	0.1636

4th drop: Beh

EFFECT				
Fixed effects	**Value**	**SE**	**t-value**	***P*-value**
(Intercept)	-0.02462	0.23626	-0.1042167	0.917
Locality	0.62682	0.32914	1.9044101	0.0572
Group size	-0.01252	0.01111	-1.1266386	0.2603
**Locality : Group size**	**-0.05636**	**0.03388**	**-1.663708**	**0.0966**
Random effects	**SD** (Intercept)	Residual		
Group	0.6341	1.2102		
**ANOVA between models**				
**Term dropped**	**df**	**AIC**	**Likelihood ratio test**	***P*-value**
3rd drop model	8	2731.220	-1357.610	
4th drop model	6	2728.413	-1358.206	0.5507

5th drop: Group size : Locality

EFFECT				
Fixed effects	**Value**	**SE**	**t-value**	***P*-value**
(Intercept)	0.08400	0.23024853	0.3648155	0.7154
**Locality**	**0.17540**	**0.17890863**	**0.9804052**	**0.3272**
Group size	-0.01961	0.01057582	-1.8543438	0.0641
Random effects	**SD** (Intercept)	Residual		
Group	0.6611	1.2094		
**ANOVA between models**				
**Term dropped**	**df**	**AIC**	**Likelihood ratio test**	***P*-value**
4th drop model	6	2728.413	-1358.206	
5th drop model	5	2729.119	-1359.560	0.0999

6th drop: Locality

EFFECT				
Fixed effects	**Value**	**SE**	**t-value**	***P*-value**
(Intercept)	0.26054	0.13589	1.91733	0.0556
Group size	-0.02534	0.00831	-3.04857	0.0024
Random effects	**SD** (Intercept)	Residual		
Group	0.6442	1.2119		
**ANOVA between models**				
**Term dropped**	**df**	**AIC**	**Likelihood ratio test**	***P*-value**
5th drop model	5	2729.119	-1359.560	
6th drop model	4	2728.053	-1360.026	0.334

Anova between the full model and the best model

ANOVA between models				
**Term dropped**	**df**	**AIC**	**Likelihood ratio test**	***P*-value**
<None> (Full model)	12	2733.438	-1354.719	
6th drop model	4	2728.053	-1360.026	0.2245

Model Validation

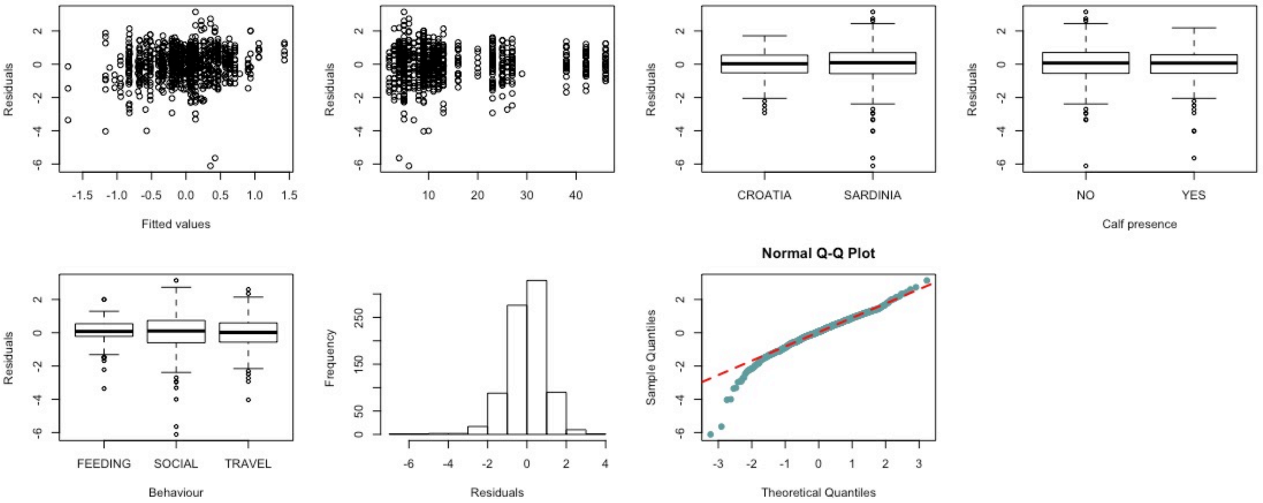

Supplement: Supplementary file 1 [file ECE3-10-1971-s001.pdf]
